# Impact of gut microbiota on the fly's germ line

**DOI:** 10.1038/ncomms11280

**Published:** 2016-04-15

**Authors:** Michael Elgart, Shay Stern, Orit Salton, Yulia Gnainsky, Yael Heifetz, Yoav Soen

**Affiliations:** 1 Department of Biological Chemistry, Weizmann Institute of Science, Rehovot 76100, Israel; 2 Department of Entomology, The Hebrew University, Rehovot 76100, Israel

## Abstract

Unlike vertically transmitted endosymbionts, which have broad effects on their host's germ line, the extracellular gut microbiota is transmitted horizontally and is not known to influence the germ line. Here we provide evidence supporting the influence of these gut bacteria on the germ line of *Drosophila melanogaster*. Removal of the gut bacteria represses oogenesis, expedites maternal-to-zygotic-transition in the offspring and unmasks hidden phenotypic variation in mutants. We further show that the main impact on oogenesis is linked to the lack of gut *Acetobacter* species, and we identify the *Drosophila Aldehyde dehydrogenase* (*Aldh*) gene as an apparent mediator of repressed oogenesis in *Acetobacter*-depleted flies. The finding of interactions between the gut microbiota and the germ line has implications for reproduction, developmental robustness and adaptation.

## Abstract (web summary)

The gut microbiota can play various roles in the host's physiology, but is not known to influence the germ line. Here, Elgart *et al*. show that certain extracellular gut bacteria can affect oogenesis and embryo development in the fruit fly.

## Graphical abstract

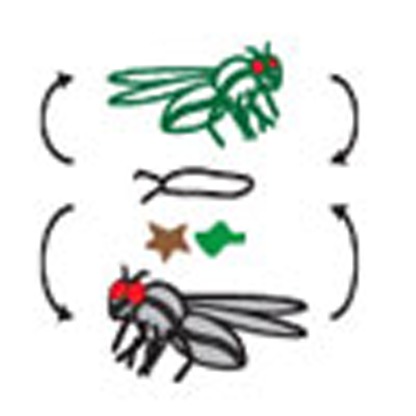

The microbiome of animals is composed of extracellular bacteria and/or endosymbionts which often take part in the development, homoeostasis, immunity and evolution of their host^1^,^2^. The activities of the extracellular gut bacteria in flies have been mainly studied in the model system *Drosophila melanogaster*^3^,^4^. The gut bacteria in *D. melanogaster* vary between strains in the wild and in the lab^4^,^5^ and can be influenced by host-intrinsic and environmental factors^6^,^7^,^8^. Laboratory stocks of *D. melanogaster* are colonized primarily by extracellular *Acetobacter* and *Lactobacillus* species^9^ which influence a broad range of somatic host functions, including growth and renewal^8^,^10^,^11^, immunity^6^,^12^,^13^, nutritional regulation^14^,^15^,^16^, mating preference^17^ and lifespan^18^,^19^ (although not in all conditions^13^). Many lab stocks are also infected with the endosymbiont *Wolbachia pipientis*^20^,^21^, which is transmitted vertically within the germ line and has been implicated in manipulation of reproduction in many *Drosophila* species^22^,^23^,^24^. Extracellular gut bacteria, on the other hand, are transmitted horizontally^25^ and have not yet been shown to have a clear impact on the germ line and reproduction. Previous work in olive fruit fly (*Bactrocera oleae*) showed that bacterial depletion by antibiotic treatment reduces fecundity in a manner that depends on the nutritional status of the flies^26^. Later work in *D. melanogaster* under rich diet conditions attributed a reduction in fecundity to the direct impact of the antibiotic on the host (as opposed to indirect effect due to bacterial loss)^14^,^27^. Analysis of antibiotic-independent effects under standard diet settings provided indirect evidence which could suggest an influence of gut bacteria on the germ line^28^. However, a conclusive statement was missing due to lack of direct evidence for microbiome influence on the state or function of reproductive tissues.

Here we provide multiple lines of evidence supporting the influence of extracellular gut bacteria (primarily gut *Acetobacter*) on oogenesis and subsequent embryonic development in the following generation. We identify the *Drosophila Aldehyde dehydrogenase* (*Aldh*) gene as an apparent mediator of repressed oogenesis in *Acetobacter*-depleted flies. Analysis of embryos of bacterial-depleted flies reveals expedited maternal-to-zygotic-transition (MZT) and overall faster embryonic development compared with control embryos. We also find that removal of extracellular gut bacteria in one generation leads to phenotypic unmasking of genetic mutations and reduces antibiotic tolerance in the next generation. Collectively, these findings uncover a hitherto unrealized dimension of gut microbiome–germline interactions.

## Results

### Loss of gut *Acetobacter* suppresses oogenesis

**Figure 1:**
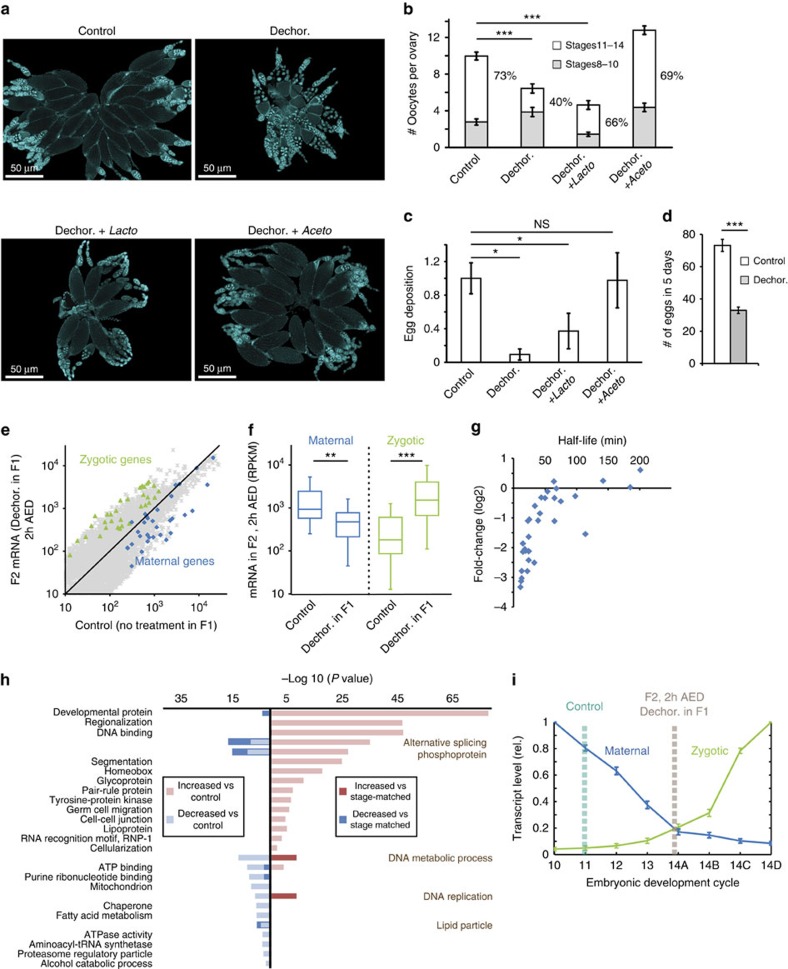
Lack of gut microbiota represses oogenesis and alters early embryonic development in the next generation. (**a**) Representative images of DAPI-stained ovaries at day 6 after eclosion, shown for untreated case (control) and for females developed from dechorionated eggs that were placed on food without bacteria (Dechor.) and food supplemented with single species of native *Acetobacter* (Dechor.+*Aceto*) or *Lactobacillus* (Dechor.+*Lacto*). Note the reduction in the size of the ovary and the number of mature oocytes without extracellular gut bacteria. (**b**) Number of oocytes per ovary and percentages of oocytes at stages 8–10 and 11–14 for the cases in **a**. Mean±s.e. based on three replicated experiments each with >20 ovaries. (**c**) Egg deposition over 4 h period relative to control. Mean fold-change ±s.e. based on three replicated experiments, each containing >20 females. (**d**) Total number of eggs deposited per female over a period of 5 days. Mean±s.e. based on three replicates, each with *n*>7. (**e**) Scatter plot of transcript levels (RPKM) in embryos of bacterial-depleted flies (Dechor.) versus control embryos (both at 2 h after egg deposition, ‘AED'). Each point represents average transcript levels based on 6 RNA-seq analyses per condition (three wild-type strains, each measured in duplicates). Blue and green overlays display maternal and zygotic transcripts selected based on Thomsen *et al*.^29^ and De-Renzis *et al*.^31^, respectively. (**f**) Global reduction in the levels of maternal transcripts and reciprocal increase in zygotic transcripts in 2 h AED embryos of bacterial-depleted flies versus control embryos. Maternal and zygotic transcripts were defined based on Lott *et al*.^30^. (**g**) Correlation between the half-life of maternal transcripts (based on ref. 29) and their extent of reduction in bacterial-depleted (Decor.) versus control embryos. (**h**) Representative enrichments of Gene Ontology (GO) annotations in groups of transcripts that were differentially expressed in 2 h AED embryos of bacterial-depleted flies compared to control embryos (pink and light blue) or to a stage-matched transcriptome (red and blue). Enrichment is represented by −log10 of (Benjamini corrected) *P* value. A more comprehensive account is provided in Supplementary Data 3 and 4. (**i**) Estimation of the developmental stage of 2 h AED embryos of bacterial-depleted flies (Decor.) and control embryos, based on transcriptome mapping to published time course data^30^. Blue and green traces display the average time course for sets of Maternal and Zygotic transcripts listed in Supplementary Data 2. Estimation and normalization was based on Efroni *et al*.^39^ using maternal genes as a reference. *P<0.05, ** *P*<0.01, *** *P*<0.001 (Student's *t*-test).

We investigated the influence of extracellular gut bacteria on reproductive capacity of the fly by eliminating the bacteria using egg dechorionation and sterilization^18^. This led to substantial changes in the ovary (Fig. 1a,b), which included reduction in the number of oocytes per ovary and in the fraction of late-stage oocytes (Fig. 1b; Supplementary Fig. 1A). These changes were consistent with a strong reduction in egg deposition (Fig. 1c,d; Supplementary Fig. 1B). Similar results were observed in a *Wolbachia*-free fly strain (Oregon R), indicating that the repression of oogenesis and reproduction in bacterial-depleted flies is independent of *Wolbachia* (Supplementary Fig. 1A,B). Notably, the reduction in egg deposition did not compromise survival to adulthood of the deposited eggs (Supplementary Fig. 1C). Successful re-colonization of the larval gut with bacteria from an isolated *Acetobacter* species, *Colony 1* (ref. 28) (Supplementary Fig. 1D), completely restored the oogenesis phenotypes (Fig. 1a–c). Similar rescue was observed when these bacteria were introduced in the adult stage (Supplementary Fig. 1E), indicating that the suppression of oogenesis in bacterial-depleted flies is reversible at any time and does not reflect irreversible failure of development. Recolonization of isolated *Lactobacillus* (*Colony 7*)^28^, on the other hand, led only to a partial rescue; it prevented the reduction in oocyte maturation, but not in the total number of eggs per ovary and egg deposition (Fig. 1b,c). qPCR-based analysis of bacterial content in the ovary of females with intact gut bacteria showed that *Acetobacter* and *Lactobacillus* spp. are not present in the ovary (Supplementary Fig. 1F), indicating that removal of gut bacteria impacts oogenesis from a remote location.

### Lack of gut bacteria expedites MZT and embryonic development

To determine if the impact of bacterial loss on the ovary and oogenesis can influence subsequent embryonic development, we analysed embryos of bacterial-depleted flies collected 2 h after egg deposition (AED). RNA-seq-based profiling of mRNA in three different fly strains (yellow white, Oregon-R and Canton-S) revealed substantial genome-wide differences in transcript levels compared with embryos of flies with intact bacteria (Supplementary Data 1). In particular, we noticed global reduction in classical maternal-effect genes^29^,^30^ and reciprocal increase in transcripts of zygotic genes^30^,^31^ (Fig. 1e,f; Supplementary Fig. 2A), including gap genes, pair-rule genes and Hox genes (Supplementary Fig. 2B–D). The increase in zygotic transcripts was accompanied by higher levels of the MZT regulator, *Zelda*^32^,^33^,^34^ and the cellularization genes, *slam*^35^, *pbl*^36^, *sry-α* (ref. 37) and *nullo*^37^,^38^ (Supplementary Fig. 2E). The global signature of reduced maternal transcripts and increased zygotic transcripts suggested that 2 h AED embryos of bacterial-depleted flies are more progressed in their development compared with embryos of flies with intact bacteria. This was also supported by a strong inverse correlation between the extent of reduction in maternal transcripts and the reported half-lives of these transcripts^29^ (Fig. 1g).

Gene ontology analysis of transcriptional differences between embryos of bacterial-depleted flies and control embryos further revealed strong enrichment of functional annotations that are consistent with a more advanced stage of development (Fig. 1h; Supplementary Data 3). In particular, the set of elevated transcripts was strongly enriched with developmental functions performed by zygotic genes (Fig. 1h, pink). Conversely, the set of reduced transcripts was enriched for annotations consistent with the expected slow-down of nuclear divisions and reduction of various metabolic functions (Fig. 1h, light blue), including breakdown of carbohydrates and lipids (Supplementary Fig. 2F,G). To evaluate influences of bacterial removal beyond the strong effect on staging, we sought to compare the transcriptome of embryos of bacterial-depleted flies with a ‘stage-matched' transcriptome. To identify the matched stage, we analysed the overlap between the transcriptome of our embryos and published transcriptomes from a time course experiment^30^. We then estimated the staging by identifying the transcriptomes which exhibit the highest overlap to our samples (as in Efroni *et al*.^39^ with emphasis on a compiled set of monotonically decreasing maternal genes; Supplementary Data 2; Supplementary Methods). Consistent with published data^40^, the transcriptome of control embryos at 2 h AED was mapped to division cycle 11 (Fig. 1i). Embryos of bacterial-depleted flies, on the other hand, were mapped close to cycle 14A, corresponding to a time difference of 30–40 min (refs 41, 42). Subsequently, we used the transcriptome of cycle 14A as a ‘stage-matched' reference for the embryos of bacterial-depleted flies at 2 h AED. Comparing the transcriptome of these embryos with the ‘stage-matched' transcriptome eliminated most of the functional enrichments (Fig. 1h, blue and red; Supplementary Data 4). The few remaining or newly appearing annotations (for example, lipid particle, alternative splicing, phosphoprotein and DNA replication) likely represent staging-independent influence of bacterial removal in the preceding generation. Together, this indicates that the advanced developmental stage accounts for most but not all of the changes in embryos of bacterial-depleted flies at 2 h AED.

**Figure 2:**
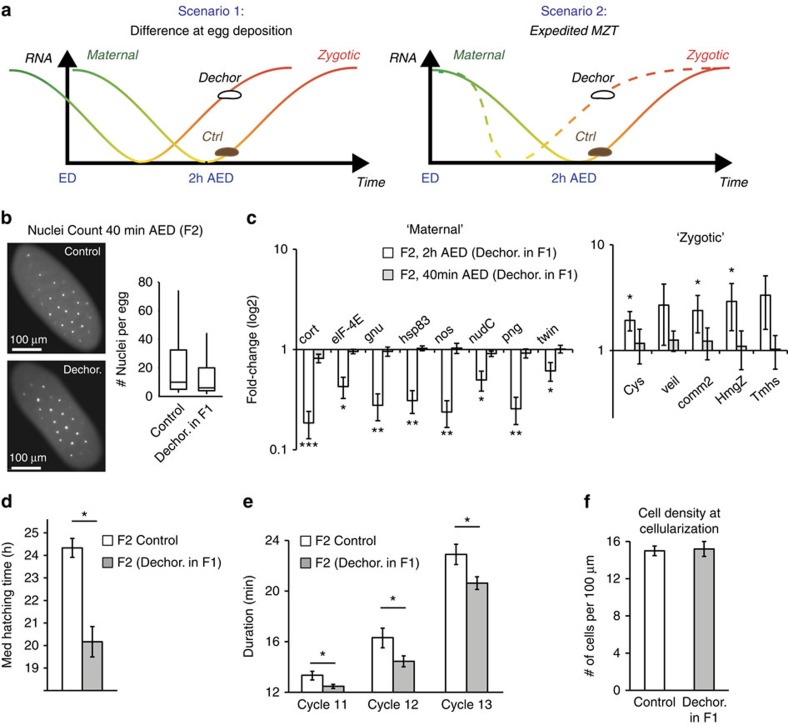
Expedited maternal-to-zygotic-transition and faster development of embryos of bacterial-depleted flies. (**a**) Two alternative scenarios that can explain global reduction in maternal RNA and reciprocal increase in zygotic transcripts. (**b**) Representative images of DAPI-stained embryos at 40 min AED (left) and the respective numbers of nuclei per embryo (right), shown for embryos of bacterial-depleted (Dechor. in F1) and control embryos. (**c**) Transcript fold-change of representative maternal and zygotic genes measured by qPCR in embryos of bacterial-depleted flies at 40 min and 2 h AED. Mean fold-change±s.e. relative to embryos of untreated flies at the respective time. Based on three biological replicates. (**d**) Effect of bacterial removal by dechorionation on the time of hatching in the next generation. Median time±s.e., based on three replicated experiments, each with >20 embryos. (**e**) Average duration of nuclear division cycles 11, 12 and 13 in embryos of bacterial-depleted flies and control embryos. Mean±s.e., *n*≥6 time courses for each case. (**f**) Density of cells at embryo mid-section immediately following the onset of cellularization. Mean number of cells per 100 μm±s.e., *n*≥5 time courses. * *P*<0.05, ** *P*<0.01 (Student's *t*-test).

The more progressed stage of development of embryos of bacterial-depleted flies at 2 h AED could reflect initial difference at the time of egg deposition or, alternatively, faster transition to zygotic transcription (Fig. 2a). To determine which of these scenarios is more likely, we analysed embryos at an earlier stage of development. Analysis of 4,6-diamidino-2-phenylindole (DAPI)-stained embryos revealed that the number of nuclei in 40 min AED embryos of bacterial-depleted flies does not exceed the number in embryos of intact flies (Fig. 2b). Lack of initial staging difference was further supported by indistinguishable mRNA levels of representative maternal and zygotic genes at 40 min AED (Fig. 2c). The clear staging difference at 2 h but not at 40 min AED indicated that the embryos of bacterial-depleted flies likely undergo expedited maternal-to-zygotic transition compared with embryos of intact flies. This conclusion was highly consistent with analysis of overall embryonic duration, which showed that the median time of hatching of embryos of bacterial-depleted flies is 3 h shorter compared with control embryos (Fig. 2d; Supplementary Movie 1). The gradual increase in staging (no detectable change at 40 min AED, 30–40 min difference at 2 h AED and 3 h difference at hatching) indicate that embryos of bacterial-depleted flies develop faster than control embryos. To determine how this expedited development is correlated with nuclear divisions in the pre-cellularization embryo, we monitored the division cycles 11–13 by time-lapse confocal microscopy applied to *His2Av-mRFP*-tagged embryos. Cycle time measurement revealed shorter durations in embryos of flies that were depleted of their gut bacteria (Fig. 2e; Supplementary Movie 2; Supplementary Fig. 5). While providing additional indication for expedited development, the estimated time difference based on cycle durations appeared to be smaller than the estimation by the genome-wide transcript profile. Further analysis of cell density at the onset of cellularization showed that the overall number of nuclear divisions is not affected by the expedited development, as indicated by indistinguishable cell densities (Fig. 2f). Survival to adulthood of embryos of bacterial-depleted flies was also unaffected (Supplementary Fig. 1C), demonstrating ability to adjust the rate of embryonic development in a non-deleterious manner.

To identify species of extracellular gut bacteria which could prevent the transcriptional differences in the embryo, we dechorionated eggs and re-introduced defined *Acetobacter* or *Lactobacillus* species. We then tested the effect of these bacterial re-introductions on the following generation of embryos at 2 h AED. Re-introduction of gut *Acetobacter* prevented the reduced mRNA levels of maternal genes in next generation of embryos (Supplementary Fig. 3A), but not the increased expression of zygotic genes (Supplementary Fig. 3B). *Lactobacillus spp*., on the other hand, could not prevent the reduced levels of maternal RNA (Supplementary Fig. 3C,D). This shows that loss of gut *Acetobacter* has a clear impact on embryogenesis in the following generation (in addition to the repressive effect on oogenesis in the first generation).

### Loss of *Acetobacter* suppresses oogenesis by repressing *Aldh*

**Figure 3:**
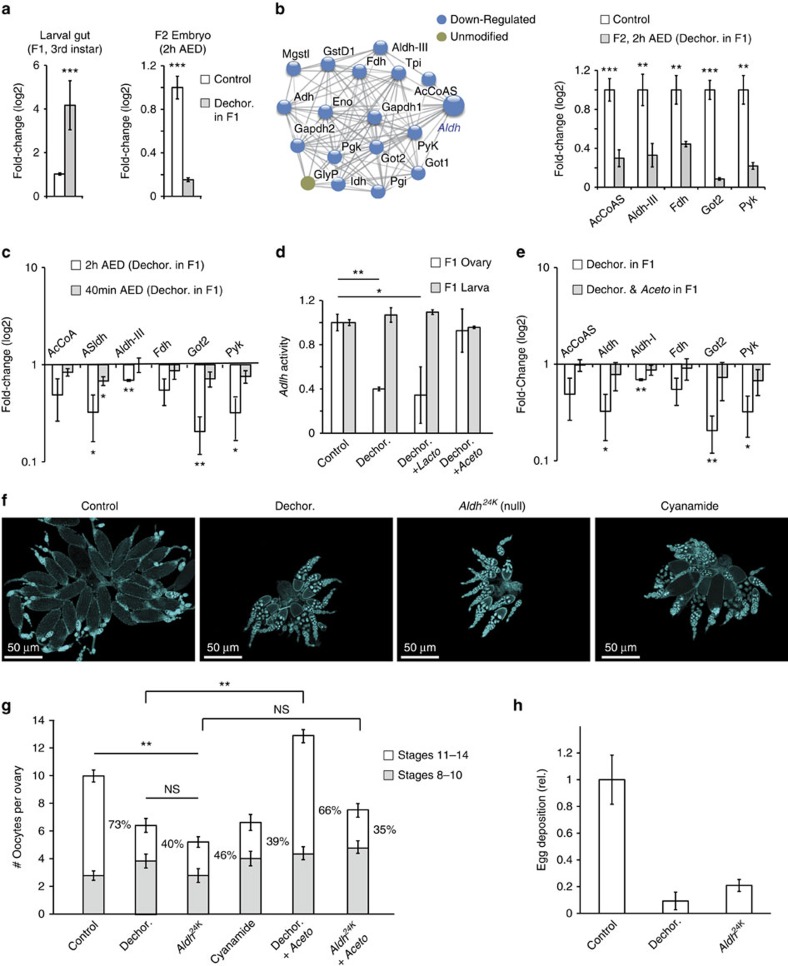
Lack of *Acetobacter* appears to suppress oogenesis by repression of *Aldh* in the ovary. (**a**) Removal of gut bacteria by dechorionation leads to tissue specific changes of *Aldh* expression in the larval gut (F1 3rd instar) and in the next generation of embryos (F2, 2 h AED). Mean fold-change (qPCR-based)±s.e. *n*=3, *** *P*<0.001 (Student's *t*-test). (**b**) RNA-seq measurements of changes in representative genes within the *Aldh* network (left), compiled using the STRING protein–protein interaction database. Blue labels designate genes that are downregulated in 2 h AED embryos of bacterial-depleted flies versus control. Mean fold-change±s.e. based on duplicates for three lines (six samples per condition). **P<0.01, ***P<0.001 (Wald Statistics, DESeq package). (**c**) qPCR-based changes in the levels of *Aldh* network genes in 40 min and 2 h AED embryos of bacterial-depleted flies relative to embryos of untreated flies at the respective time. Mean fold-change±s.e. *n*=3, *P<0.05, ** *P*<0.01 (Student's *t*-test). (**d**) Enzymatic activity of Aldh in the gut of 3rd instar larvae and ovary of 6-day-old adult females. Shown are data for intact females ('Control') and females that were developed from dechorionated eggs ('Dechor.'), with and without prior re-introduction of *Actetobacter* or *Lactobacillus* species. Mean fold-change versus control±s.e., *n*≥3, *P<0.05, **P<0.01 (Student's *t*-test). (**e**) qPCR-based changes in the levels of *Aldh* network genes in 2 h AED (F2) embryos of bacterial-depleted (F1) flies, with and without re-introduction of *Actetobacter* species in F1. Mean fold-change versus control±s.e., *n*≥3, *P<0.05, **P<0.01 (Student's *t*-test). (**f**) Representative images of DAPI-stained ovaries at day 6 after eclosion of untreated females ('Control') and females developed from dechorionated eggs (Dechor.), *Aldh* null egg ('*Aldh*^*24K*^ (null)') and wild type females in which *Aldh* has been inhibited by exposure to cyanamide throughout the larval and adult stage ('Cynamide'). Note the similar impact of bacterial removal and *Aldh* loss (or inhibition) on ovary size and the number of mature oocytes. (**g**) Number of oocytes per ovary and percentages of oocytes in stages 8–10 and 11–14. Data corresponds to the cases in **f** and to wild-type and *Aldh*^*24K*^ null females developed after dechorionation and re-introduction of defined *Acetobacter* spp. (‘Dechor.+*Aceto*' and ‘*Aldh*^*24K*^+*Aceto*', respectively). Mean±s.e. based on three replicated experiments, each with >20 ovaries. **P<0.01 (Student's *t*-test). (**h**) Relative egg deposition by 6-day-old *Aldh* null females, wild type females and females that were developed from Dechorionated eggs. Mean fold-change compared to control±s.e. based on four replicated experiments, each with five vials. **P<0.01 (Student's *t*-test).

We have previously found that larval exposure to the aminoglycoside antibiotic, G418, leads to selective depletion of gut *Acetobacter*, which persists in the non-exposed offspring^28^. In addition to this heritable change in microbiome composition, exposure to G418 led to heritable induction of Drosophila *Aldh* in the larval foregut^43^. Since antibiotics also have bacterial-independent effects on the host tissue^27^,^28^, we tested if the change in *Aldh* is indeed caused by *Acetobacter* depletion. Analysis of *Aldh* expression after removal of gut bacteria by egg dechorionation revealed tissue-specific effects which partly (but not fully) overlap with the effects of G418. Similarly to G418 treatment, dechorionation upregulated *Aldh* in the gut of 3rd-instar larvae (Fig. 3a, left). However, unlike G418, dechorionation led to downregulation of *Aldh* in the following generation of embryos (Fig. 3a, right). This reduction of *Aldh* mRNA in the embryos was accompanied by downregulation of almost all the closely related (‘*Aldh* network') genes that we compiled using the STRING database^44^ (Fig. 3b; Supplementary Fig. 4A). A smaller reduction in *Aldh* mRNA was also observed at 40 min AED (Fig. 3c), suggesting that this reduction is independent of the general decrease of maternal transcripts. This suggestion was further confirmed by analysis of *Aldh* enzymatic activity which revealed substantial reduction of *Aldh* activity already in the ovary of bacterial-depleted females (Fig. 3d). The reduction in *Aldh* activity and the subsequent downregulation of *Aldh* mRNA in the embryos were both abolished by re-introduction of *Acetobacter* species (Fig. 3d,e). These findings indicate that depletion of gut *Acetobacter* affects *Aldh* expression in a stage- and tissue-specific manner: it upregulates *Aldh* in the larval gut but represses *Aldh* in the ovary and in the early embryos of these *Acetobacter*-free flies.

To determine if the reduction in *Aldh* expression in *Acetobacter*-depleted flies contributes to the negative impact on oogenesis, we analysed two *Aldh* null lines, *Aldh*^*17H*^ and *Aldh*^*24K*^ (ref. 45). The loss of *Aldh* did not compromise the expression of the *Aldh* network genes (Supplementary Fig. 4B), thus enabling analysis of the specific contribution of *Aldh* and its involvement in the *Acetobacter* impact. Lack of *Aldh* function phenocopied the effects of bacterial depletion in the ovary. In particular, we observed decrease in ovary size (Fig. 3f), reduced number and maturation of oocytes (Fig. 3g) and decreased egg deposition (Fig. 3h). However, unlike in wild-type flies, re-introduction of *Acetobacter* to bacterial-depleted *Aldh* null flies could not rescue the reduction in oogenesis (Fig. 3g). The effects of genetic loss-of-function of *Aldh* were reproduced by chemical inhibition of *Aldh* using cyanamide, a specific inhibitor of *Aldh* (Fig. 3f,g). Overall, these results show that: (i) lack of gut *Acetobacter* represses *Aldh* expression in the ovary, (ii) this repression of *Aldh* appears sufficient to suppress oogenesis and (iii) *Aldh* is necessary for the enhancement of oogenesis by *Acetobacter* supplementation. In contrast to the ability of *Aldh* loss-of-function to phenocopy the effect of *Acetobacter* depletion on oogenesis, loss of *Aldh* did not reproduce the mRNA changes in the embryos (Supplementary Fig. 4C,D). Thus, *Aldh* seems to mediate the impact of bacterial removal on oogenesis, but not on the embryos.

### Gut bacteria contribute to the stability of host phenotypes

**Figure 4:**
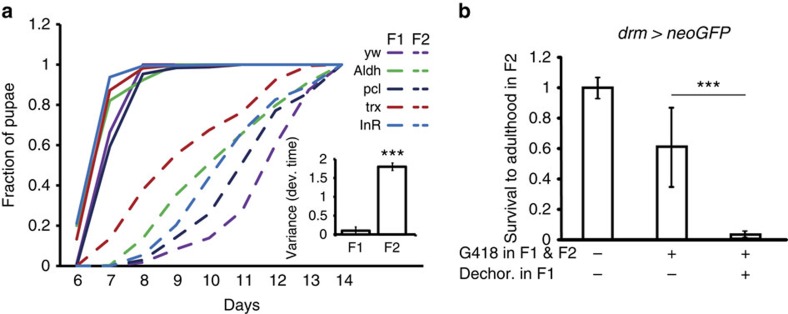
Removal of extracellular gut bacteria exposes 'hidden' mutations and reduces stress tolerance in the next generation. (**a**) Effect of removal of gut bacteria in F1 on the kinetics of pupation in F1 and F2, shown for wild-type flies (*yw*) and 4 mutant lines (InR, *trx*, *pcl* and *Aldh*). Inset: statistical analysis of the variance in pupation time due to difference in genotype. Shown are variances in genotype-specific median time to pupation. Mean variance±s.e., *n*=3. Note the substantially larger variance in F2 versus F1. ***P<0.001 (Student's *t*-test). (**b**) Effect of removal of gut bacteria in F1 on the survival of F2 *drm>neoGFP* flies that were exposed to 400 μg ml^−1^ of G418 in both generations. Note the strong reduction in the survival of F2 flies when G418 treatment was preceded by egg dechorionation in F1. Mean survival to adulthood relative to untreated control±s.e., *n*=3, **P<0.01 (Student's *t*-test).

The above analysis was performed in wild-type flies under conditions which promote robust propagation of lab strains and might therefore mask some of the impacts of bacterial depletion. We therefore evaluated functional consequences of removing bacteria on the background of genetic changes and environmental stress. In particular, we tested if loss of gut bacteria influences phenotypic stability in several fly lines carrying heterozygous mutations in the following genes: *polycomb-like* (*pcl*), *trithorax* (*trx*), *insulin receptor* (InR) and *Aldh* (not a null line). We removed gut bacteria by egg dechorionation and analysed the rate of pupation in the same generation (F1) and the following generation (F2). While none of the mutations influenced the rate of pupation in F1, they led to substantial, mutation-specific differences in the offspring generation (Fig. 4a). Thus, the lack of gut bacteria uncovered mutation-specific phenotypes in F2 that were masked in F1. The unmasking of mutations only in the second generation suggests that the removal of bacteria in F1 destabilizes embryogenesis in F2 to an extent that no longer supports phenotypic buffering of the mutations. To test if such destabilization can also affect stress tolerance without mutations, we used the toxicity model of Stern *et al*.^43^. Specifically, we generated transgenic *drm>neoGFP* flies carrying a G418-resistance gene (*neoGFP*) under the control of the drumstick (*drm*) promoter and investigated G418 tolerance in F2 larvae with and without prior removal of gut bacteria by egg dechorionation in F1. The pre-elimination of gut bacteria in F1 led to much stronger reduction in the survival of F2 offspring that were exposed to G418 in both generations (Fig. 4b). This differential stress tolerance suggests that removal of bacteria destabilizes subsequent embryogenesis to an extent which severely compromises resistance to toxic stress in F2.

## Discussion

Unlike endosymbionts, which colonize the germ line and manipulate reproductive success^22^,^24^,^46^,^47^, extracellular gut bacteria are transmitted horizontally and have so far not been directly shown to influence the germ line. We hypothesized, however, that gut bacteria can modify somatic factors, which in turn influence the germ line. This could then affect embryonic development in the next generation despite the isolation between the embryo and the horizontally transmitted bacteria^48^. Here we provide evidence for such influences, based on the effects of gut bacteria on oogenesis, fecundity and embryogenesis. While previous (antibiotic-based) evaluations did not reveal bacterial influence on fecundity^14^,^27^, it is possible that the lack of influence on fecundity was caused by the use of a rich diet. This is further supported by the ability to prevent reduction in fecundity by increasing the concentration of yeast to the previously reported levels^14^ (Supplementary Fig. 1G). It is also consistent with diet-dependent prevention of various other phenotypes of bacterial removal^11^,^12^,^29^,^31^.

The suppression of oogenesis and fecundity in the current study is mainly linked to the lack of *Acetobacter* species and appears to be mediated by *Drosophila Aldh*. Specifically, we show that lack of gut *Acetobacter* (but not *Lactobacillus*) reduces the activity of *Aldh* in the ovary (but not in the gut) and reduces *Aldh* levels in the early offspring embryo. We also show that loss (or inhibition) of *Aldh* function, is sufficient to phenocopy the effect of *Acetobacter* depletion on oogenesis and that *Aldh* is necessary for the *Acetobacter*–mediated rescue of oogenesis. These findings extend the scope of *Aldh* influences in *Drosophila*, beyond the previously reported contributions to ethanol detoxification^49^,^50^,^51^, survival under hyperoxia and resistance to starvation^45^. Since we did not include ethanol in the fly's diet, the contribution of *Aldh* to oogenesis is likely mediated by ethanol-independent activities of *Aldh*. One activity that might be of particular relevance is the breakdown of many other types of endogenous and exogenous aldehyde substrates in both eukaryotes and prokaryotes^52^. These include substrates (for examples, glyceraldehydes) derived from common sugars in the fly's food, such as fructose and glucose. This suggestion is consistent with detoxification of (peroxidation-induced) reactive aldehydes^45^ and with the contribution of aldehyde dehydrogenase type III to dietary sugar tolerance in *Drosophila*^53^. The upregulation of *Aldh* in the gut of bacterial-depleted larvae (as opposed to downregulation in the adult ovary) further suggests that *Aldh* mediates additional activities in the gut. Nevertheless, further research is required to elucidate the mechanisms by which *Acetobacter* modulates *Aldh* expression, and how this in turn affects oogenesis.

The influence of the extracellular gut microbiota on the germ line could mediate trans-generational effects on the host. A particularly surprising effect of *Acetobacter* depletion was the expedited MZT and faster embryonic development. The timing of MZT is known to vary between species^40^,^54^. It has also been shown to depend on nuclear-to-cytoplasmic ratio^55^,^56^,^57^ and on regulatory genes, such as Zelda^32^,^33^,^34^, grapes^56^ RpII215 (ref. 58) and the Cdc25 phosphatases, *string* and *twine*^57^,^59^. Collectively, these genes affect *Drosophila* MZT 32,33,56,57,58,59, the number of nuclear divisions before cellularization^58^,^60^ and the duration of mitotic cycles^55^,^58^. Certain genetic disruptions of these effectors have also been shown to cause deleterious effects on embryogenesis^32^,^58^. However, the ability to alter MZT and pre-cellularization development by environmental-like changes (and the consequences of these changes) has not yet been explored. Here we show that removal of extracellular gut microbiota in one generation can expedite MZT and increase the rate of pre- and post-cellularization development in the following generation (Fig. 2b–e). The pre-cellularization influence was manifested by the pattern of global changes in maternal and zygotic transcripts and by shortening of nuclei division cycle lengths. While both of these independent analyses supported expedited development of embryos of bacterial-depleted flies, the transcription-based estimation revealed a larger difference compared with the cycle length-based estimation (30–40 min versus ∼10 min staging difference at 2 h AED, respectively). This discrepancy could potentially reflect limited time resolution of the transcription-based estimation and/or imperfect synchronization between the ‘transcriptional stage' and nuclei status. The extent by which the MZT kinetics varies across different choices of fly lines and rearing conditions is not known. Such variation does not confound the finding of faster development of embryos of bacterial-depleted flies (versus control), because these embryos differ only in the bacterial content of their parents. Nonetheless, variations in the MZT kinetics across different lines and rearing conditions would modify the transcriptional-based staging and could potentially lead to over-estimation of the staging difference. Additionally, we cannot exclude the possibility that deficiencies in the fertilized eggs of bacterial-depleted flies may compromise the precision of coordination between transcriptional changes and nuclei division status. In this hypothetical scenario, both estimations might be correct because the ‘transcriptional stage' does not need to precisely match the ‘division cycle stage'. Given these limitations on stage estimation, it would be safer to assume that the difference between 2 h embryos of bacterial-depleted flies versus controls is only about 10 min. This would also be easier to reconcile with the lack of change in total number of nuclear divisions and the non-deleterious impact of adjusting the rate development in a critical phase.

The faster development of embryos of bacterial-depleted flies is followed by a substantial delay in larval development, which is not observed in the first generation without bacteria (Fig. 4a). How the lack of extracellular bacteria in one generation leads to faster embryogenesis and then to slower larval development is not clear. Based on the dependence of MZT on the nuclear-to-cytoplasmic (N/C) ratio, one might posits that loss of gut microbiome leads to cytoplasmic deficiencies in maternally deposited factors, which in turn increases the effective N/C ratio and expedites zygotic activation. Increasing the rate of embryonic development may, in turn, cause damage which induces stress during subsequent stages of larval development. Combined with the lack of gut bacteria, this stress could be responsible for a substantial delay in larval development, which is not observed in the first generation.

The above interpretation of altered development and stress in the absence of gut bacteria may also account for the unmasking of phenotypes in mutant backgrounds (Fig. 4a) and the reduced tolerance of toxic load (Fig. 4b). It has been previously argued that participation of co-evolving symbionts in a wide range of host processes^1^,^2^ makes the stability of these processes dependent on the symbionts^61^,^62^. This is analogous to buffering by host-intrinsic mechanisms which influence a wide range of targets, such as *Hsp90* (refs 63, 64, 65), *Polycomb*^43^,^66^ and microRNAs^67^,^68^,^69^. The negative impact of bacterial removal on the robustness of its host provides evidence for extension of host-intrinsic buffering to stabilization by functions and/or products of the gut microbiome. The proposed involvement of the gut microbiome in phenotypic buffering is consistent with recent work in flies demonstrating among-genome variations in the impact of bacterial removal on five host nutritional indices (weight, protein, lipid, glucose and glycogen contents)^16^. It is also in line with substantial evidence in other organisms in which microbial disruptions are associated with a wide range of disease states^70^,^71^,^72^,^73^,^74^,^75^. The possibility for inheriting environmentally induced changes in the microbiome^28^ further provides a substantial potential for transgenerational influences on developmental stability, disease susceptibility and adaptability.

## Methods

### *Drosophila* stocks

*D. melanogaster Yw*, *OrR*, *CanS*, *trx[1]*, *pcl* [s1859], *Aldh* [KG02748], *InR* [93Dj-4], His2Av-mRFP1 and *drm*-GAL4 lines were obtained from the Bloomington Stock Center. For the experiments involving multi-generational stress tolerance in the presence/absence of bacteria, the GAL4 driver with the *UAS-neoGFP* transgene was used as described^43^. The *Aldh* null lines, *Aldh*^*17H*^ and *Aldh*^*24k*^ were kindly provided by James D. Fry (the University of Rochester).

### Food preparation

Standard cornmeal food was used as the base for all the experiments and was prepared as described in the Bloomington Stock Center recipe (http://flystocks.bio.indiana.edu/Fly_Work/media-recipes/molassesfood.htm). To maintain sterile conditions after dechorionation, all plasticware and caps were used after ultraviolet sterilization and the work was performed in a biological hood. Boiling hot food was aseptically transferred to the hood and allowed to cool. Propionic acid and methylparaben were added (as specified in the recipe) and the food was dispensed (in 10-ml aliquots) into ultraviolet-sterilized plastic vials (25 × 95 mm; cat# 51-0500, Biologix, USA) or bottles.

For experiments with *Aldh* inhibitor or G418, warm food (∼60 °C) was mixed with either Cyanamide (Sigma) or water-dissolved G418 so as to reach final concentrations of 100 μM for Cyanamide and 400 μM for G418.

### Dechorionation experiments

Embryos were collected and dechorionated for 2 min in 2.7% sodium hypochlorite solution, then washed twice in 70% ethanol and then twice with sterile, distilled water as was described by Brummel^18^,^25^. Embryos were then transferred to sterile food and allowed to develop. Ethanol-based protocol was chosen to ensure complete removal of extracellular bacteria and to assist the prevention of contamination in the absence of antibiotics (the risk of contamination appears to increase significantly when the bacteria are removed without antibiotics). The ethanol step was avoided in some of the experiments (Supplementary Fig. 1A) to exclude the possibility that it might influence the main findings. To further reduce the likelihood of contamination, all manipulations of the bacterial-depleted flies were performed in a biological hood. Specifically the embryos were collected and dechorionated on the bench, but were rinsed in ethanol and immediately transferred to the sterile hood, where the second ethanol wash and the two sterile water washes were performed. Additionally, vials and bottles containing dechorionated flies were monitored for the characteristic developmental delay phenotypes of bacterial-depleted flies. Vials and bottle that were suspected of contamination, were inspected using LB growth assays or by qPCR with 16S probes (see below). For the transgenerational experiments, F1 flies that were developed from these dechorionated embryos were collected after 17–20 days from the start of the experiment (4–7-day-old adults) and the same number of males and females were allowed to mate again in vials.

### Supplementing fly food with bacteria

For supplementing the fly food with bacteria, *Acetobacter* from *Colony 1* and *Lactobacillus* from *Colony 7* (ref. 28) were cultured at 30 °C to 1 OD_600_ (∼10^8^ cells per ml). A final volume of 100 μl was applied directly to the top of the fly food.

### Measuring egg deposition and survival to adulthood

*Egg deposition*. For the short-term egg deposition experiments, five males and five females were placed in each vial, synchronized twice with 1-h interval and transferred to a new vial. Flies were then allowed to lay eggs for 4 h and the number of deposited eggs was counted. Each condition (that is, dechorionation with or without re-introduction of specific bacterial species) was measured in three biological repeats, each consisting of 10 vials. For the longer-term egg deposition (fecundity) experiments, two males and one female were placed in each vial, synchronized twice with 1-h interval and moved to a new vial. Flies were then transferred to a new vial every 24 h for 5 days and the number of eggs deposited in the previous day was counted.

*Survival to adulthood*. At day 13 and 15 after the start of egg deposition, we measured the number of pupae (measure for eggs that hatched) and the number of adults for each vial.

### DAPI staining and analysis of ovaries and embryos

Ovaries were dissected from 6-day-old females in Ringer's medium. They were then washed once in Ringer's medium and fixated in 5% formaldehyde solution in phosphate-buffered saline (PBS) for 20 min. The ovaries were then washed once in PBST (1% TritonX), and incubated in PBST for 1 h. The ovaries were then mounted in Vectashield mounting medium with DAPI on glass slides and imaged. For embryo staining, the eggs were dechorionated, and fixated in equal volume of heptane and 3.7% formaldehyde in PEM buffer for 20 min. The embryos were then devitellinized via shaking in methanol, and mounted in Vectashield mounting medium with DAPI on glass slides and imaged.

DAPI-stained ovaries were imaged individually and each ovary was scored for the number of oocytes at stages 8–10 and 11–14. Each of the evaluated condition (dechorionation, *Aldh* mutant, bacterial supplementation and control) consisted of at least three pooled biological repeats of 20 ovaries or more. DAPI stained embryos were scored for the number of nuclei per embryo.

### Measuring bacterial content by qPCR

To measure bacterial composition in the gut, 7–10 3rd instar larvae were collected, their gut dissected and pooled; for ovaries, 10 ovaries from 3 to 6 day old females were dissected. Bacterial genomic DNA was extracted using, chemagic DNA Bacteria kit (Chemagen), and 5 ng of purified DNA was used per qPCR reaction. All reactions were performed in triplicates on a qPCR machine (Applied Biosystems 7900HT Fast Real-Time PCR System, Life Technologies Corporation) using SYBRGreen in 384-well plates. The species-specific primers that were used: aceto_rt_1_f (5′-TAGTGGCGGACGGGTGAGTA-3′), aceto_rt_1_r (5′-AATCAAACGCAGGCTCCTCC-3′), lacto_rt_2_f (5′-AGGTAACGGCTCACCATGGC-3′), lacto_rt_2_r (5′-ATTCCCTACTGCTGCCTCCC-3′), wolb_rt_2_f (5′-CAATGGTGGCTACAATGGGC-3′), wolb_rt_2_r (5′-GTATTCACCGTGGCGTGCTG-3′).

Drosophila actin gene was used to normalize the bacterial content using the primers: dros_rt_1_f (5′-GGAAACCACGCAAATTCTCAGT-3′), dros_rt_1_r (5′-CGACAACCAGAGCAGCAACTT-3′).

### RNA extraction from eggs

About 200–300 adult flies (control or dechorionated) were synchronized for 1 h and allowed to deposit eggs for either 2 h or 40 min on a 10 cm agar plate. Eggs were collected from the plate and washed once in PBS. PBS was then removed and eggs were squashed with tissue homogenizer. Lysis buffer (400 μl) was added and the sample was flash frozen. After thawing, total RNA was cleaned using the ZR RNA MicroPrep Kit (lysis buffer was from the same kit). Samples were eluted in 10 μl double distilled water (ddw) and stored in −80 °C until use.

### RNA-seq library preparation and sequencing

The cDNA libraries were prepared from poly-A mRNA following the manufacturer's instructions in the Illumina RNA sample preparation kit. In short, poly (T) oligo-attached magnetic beads were used to purify the poly(A)-containing mRNA molecules. The mRNA was fragmented into 200–500 bp segments. RNA fragments were converted into cDNA using SuperScript II reverse transcriptase (Life Technology) and random hexamer primers. Adaptors were ligated to the cDNA fragments, followed by purification, PCR and additional purification.

Deep sequencing measurement of RNA was performed in the Genomics Core Facility unit of the Weizmann Institute of Science (Rehovot, Israel) using Illumina Genome Analyzer IIx (GA IIx). For sequencing, we used the following experimental kits and reagents: (1) Standard Cluster Generation Kit (#GD-103-4001, Illumina, San Diego, CA, USA) containing all reagents necessary to load the samples on to the flow cell and perform the bridge amplification, (2) Illumina Sequencing Kit v5 (TruSeq SBS Kit v5 GA (36-cycles), FC-104-5001) which contains the reagents for the sequencing runs, and (3) the GA IIx Sequencing Control Software version SCS 2.8, which was used to control the sequencer. Sequencing was based on 100 bp non-paired reads.

mRNA extracted from 12 samples (biological duplicates from three wild-type strains (yellow white, OregonR, Canton S), with and without extracellular gut bacteria) was barcoded in the ligation step by Illumina standard multiplex adaptors. The multiplexed samples were sequenced on a single lane to yield total ∼110 × 10^6^ reads (between 6 and 12 million reads per sample).

### Quantitative PCR analysis of RNA

Total RNA was extracted from embryos as described, and mRNA was converted to cDNA using a high-capacity reverse transcription kit (Applied Biosystems, Carlsbad, CA, USA). Transcript levels were measured using real-time quantitative PCR (qPCR) on a 7900HT Fast Real-Time PCR machine with PerfeCTa SYBR green FastMix, ROX (Quanta Biosciences, Gaithersburg, MD, USA). A total of 2.5 ng cDNA was used per reaction with three technical replicates. The representative genes were chosen based on several criteria: first a list of maternal genes (based on Thomson *et al*.^29^) and zygotic genes (based on De Renzis *et al*.^31^) was compiled; next this list was intersected with the list of genes that were differentially expressed in our experiment; then we discarded the genes that were expressed at low levels in our control and treatment groups or had inconsistent expression levels across replicates; finally, top differentially expressed genes were chosen for verification. Most of the primers used in this study were previously verified primers from DRSC FlyPrimerBank. The specific primers in this study were:

Act5C: 5′-CCCTCGTTCTTGGGAATGG-3′, 5′-CGGTGTTGGCATACAGATCCT-3′; Aldh: 5′-AGAACTTCGCAGCAGCTGTTG-3′, 5′-TGTTGATAAATACCCCGGTGTAGA-3′; cort:5′-GGGAGAACATCGAGTTCAAC-3′, 5′-CGGTCAAAGTGACACCCGT-3′; elF-4E: 5′-TGGGAGGACATGCAAAACGA-3′, 5′-GCGAGTAGTCACTACCCAGC-3′; gnu: 5′-CGGCATTGTCCGGGTTAAAAC-3′, 5′-GTACTTTGACTTGTGGGCGAA-3′; nos: 5′-CACCGCCAATTCGCTCCTTAT-3′, 5′-GCTGGTGACTCGCACTAGC-3′; nudC: 5′-CCCGAGTTCCTTGGCACTTT-3′, 5′-AGCTTCTCCCACTCCGTCT-3′; png: 5′-GGGTCTTCCTCTGCCACCAA-3′, 5′-CAACTCTGTCTTCGGATTC-3′; twin: 5′-TGCCCACATCCGCATATACC-3′, 5′-TGGCTAGCCGTATGCATCAG-3′; cys: 5′-GGATGCCACTCTCGCACAG-3′, 5′-GGTGTTAAGACTTCCAGCTACG-3′; veil: 5′-CCGATTCGAGCAGACGAGTG-3′, 5′-CGCCTCCTTGCGATACTTTC-3′; comm2: 5′-GATTCTGAACAGCGGGAGAAG-3′, 5′-TCAGGAAATCACTGCTGGAGAT-3′; HmgZ: 5′-CAGCAAGGTGACAGACATCG-3′, 5′-CCTCCTTCATTTTGATGGCCT-3′; Tmhs: 5′-CGTGGCATTCGTGACACCA-3′, 5′-GTCAGCAAGGCCAGTGCTAT-3′; AcCoAS: 5′-CCATGATTCTGGAGCTGCCTA-3′, 5′-GCCTTCAGGTACAGGGGTTTA-3′; Aldh-III: 5′-TCTACAATGACCCCTTCGGAG-3′, 5′-CGCAGACAACTGGATAGCAATC-3′; Fdh: 5′-CGCTTGGGAGGCAAAGAAAC-3′, 5′-CCAGCCTTGAAGTTGGTCAC-3′; Got2: 5′-TGTCACGGAAGCCTTCAAGAA-3′, 5′-GTCCAGACTACGGCTCACC-3′; Pyk: 5′-GGTCTTGGTGACTGGCTGAA-3′, 5′-TTCTTTCCGACCTGCAGACC-3′.

### Measuring duration of larval development

Unless indicated otherwise, delay in larval development was measured by crossing 2 females and 2–3 males for 2–3 days in vials. The number of pupae in each vial was counted daily. The integrated number of pupae that formed before each inspection time was normalized to the total number of pupae that were formed in the vial at the end of the experiment.

### Measuring hatching time

About 200–300 adult flies (control or dechorionated) were synchronized twice for 1 h and allowed to lay eggs for 30 min on a 10 cm agar plate. Eggs were collected from the plate and transferred to a small area on a 10 cm plate with fly food. Eggs (20–50) from control and dechorionated adult flies were arranged on different ends of the same plate to allow simultaneous imaging. To reduce unnecessary damage due to suboptimal conditions during pre-hatching stages, the eggs were kept in an incubator for 17 h before imaging. Magnification images (4 ×) were then taken every minute for 9 h using a LEICA M165 FC microscope equipped with Nikon digital sight DS-Fi1 camera. Hatched larvae were removed from the plate to avoid disturbance to the remaining eggs and hatching events were recorded.

### Live embryo imaging

Several hundred of *His2Av-mRFP1* adult flies (control or dechorionated) were synchronized twice for 1 h and allowed to lay eggs for 20 min on a 10 cm agar plate. Eggs were collected from the plate, washed in sterile water and dechorionated. Eggs were then transferred to MatTek Glass-Bottom Dishes that were pre-treated with embryo glue (3 M tape in Heptane) and covered in Halocarbon oil 700 (Sigma). The eggs were imaged using UPLSAPO 20 × numerical aperture:0.75 objective of the confocal OLYMPUS FV1000 microscope with temperature-controlled chamber (set at 25 °C) and IX81 ZDC Motorized Stage. Every embryo was imaged once every ∼2.5 min for a total duration of ∼4 h. The focus was maintained throughout the imaging period using the ZDC method. Image analysis was performed using ImageJ.

Cycle durations were measured as the time difference between the first frames after consecutive nuclei separation.

Cell density at cellularization was determined by the number of nuclei within a 100 μm region (at a mid-embryo optical section).

### G418 experiments

Flies were reared on food with or without G418 supplementation as described above. For experiments which did not include dechorionation, three males and two females were allowed to mate and deposit eggs for 3 days in vials. F1 flies which developed from these eggs were transferred 4–7 days after eclosion and the same number of males and females were allowed to mate again in vials. For experiments with dechorionation, 15–20 dechorionated eggs were transferred to each vial. F1 flies which developed from these eggs were transferred 4–7 days after eclosion and allowed to mate in vials (three males and two females per vial). In all the experiments, the rates of survival to adulthood were measured by counting the number of adults in F2 and dividing them by the average count for naive flies in F2.

### Additional methods

See Supplementary Methods for description of the following methods:

- RNA-seq analysis
- Estimating developmental stage based on the transcriptional profile
- Inferring the *Aldh* network
- Measuring enzymatic activity of *Aldh*

## Additional information

**Accession codes:** The RNA-seq data have been deposited in the NCBI Sequence Read Archive (SRA) database with accession codes SRP070618 and SRP070619.

**How to cite this article**: Elgart, M. *et al*. Impact of gut microbiota on the fly's germ line. *Nat. Commun.* 7:11280 doi: 10.1038/ncomms11280 (2016).

## Supplementary Material

Supplementary Information Supplementary Figures 1-5, Supplementary Methods and Supplementary References

Supplementary Movie 1 Time-lapse imaging of the final stages of embryonic development. Records correspond to wild-type (*yw*) embryos produced by bacterial-depleted flies (bottom, 'Dechor.') and control flies (top). Shown are 3 representative embryos in each case, imaged at 1min resolution from 19 to 25hrs after egg deposition (time after egg deposition is indicated in the upper left corner). Note the ~3h difference in average hatching time. 4X images were taken using a dissection microscope.

Supplementary Movie 2 Time-lapse confocal microscopy of embryonic development from the onset of mitotic division 11 to cellularization. *His2Av-mRFP1* embryos produced by control (top) and bacterial-depleted flies (bottom, 'Dechor.') were imaged every 30s. Shown are maximal intensity projection (MIP) visualizations allowing clear identification of metaphases and replication waves. The onset of cycle 11 in each embryo was identified by back tracing divisions prior to cellularization (which is easy to detect). Time from the beginning of cycle 11 is indicated in the upper left corner. Duration of each cycle is represented by colored progress bars, overlaid (with text labels) on each embryo. The accumulated difference between the durations from cycle 11 to 13 is indicated by a red bar. Note the shorter cycle durations of the embryo of bacterial-depleted flies.

Supplementary Data 1 Summary table for RNA-seq analysis of Dechorionated (2 yw + 2 OrR + 2 CanS) vs Control (2 yw + 2 OrR + 2 CanS) drosophila 2h embryos.

Supplementary Data 2 List of genes that were used as a 'ruler' when calculating development time (based on timeseries data from Lott *et al.*).

Supplementary Data 3 Gene ontology analysis of transcriptional differences between embryos of bacterial-depleted flies and control embryos.

Supplementary Data 4 Gene ontology analysis of transcriptional differences between embryos of bacterial-depleted flies and 'stage-matched' embryos.
