# Supplementary material for: Impact of gut microbiota on the fly's germ line
Source: Nat Commun. 2016 Apr 15;7:11280. doi: 10.1038/ncomms11280 (PMC4835552; doi:10.1038/ncomms11280)
Supplement: Supplementary Information — Supplementary Figures 1-5, Supplementary Methods and Supplementary References [file ncomms11280-s1.pdf]

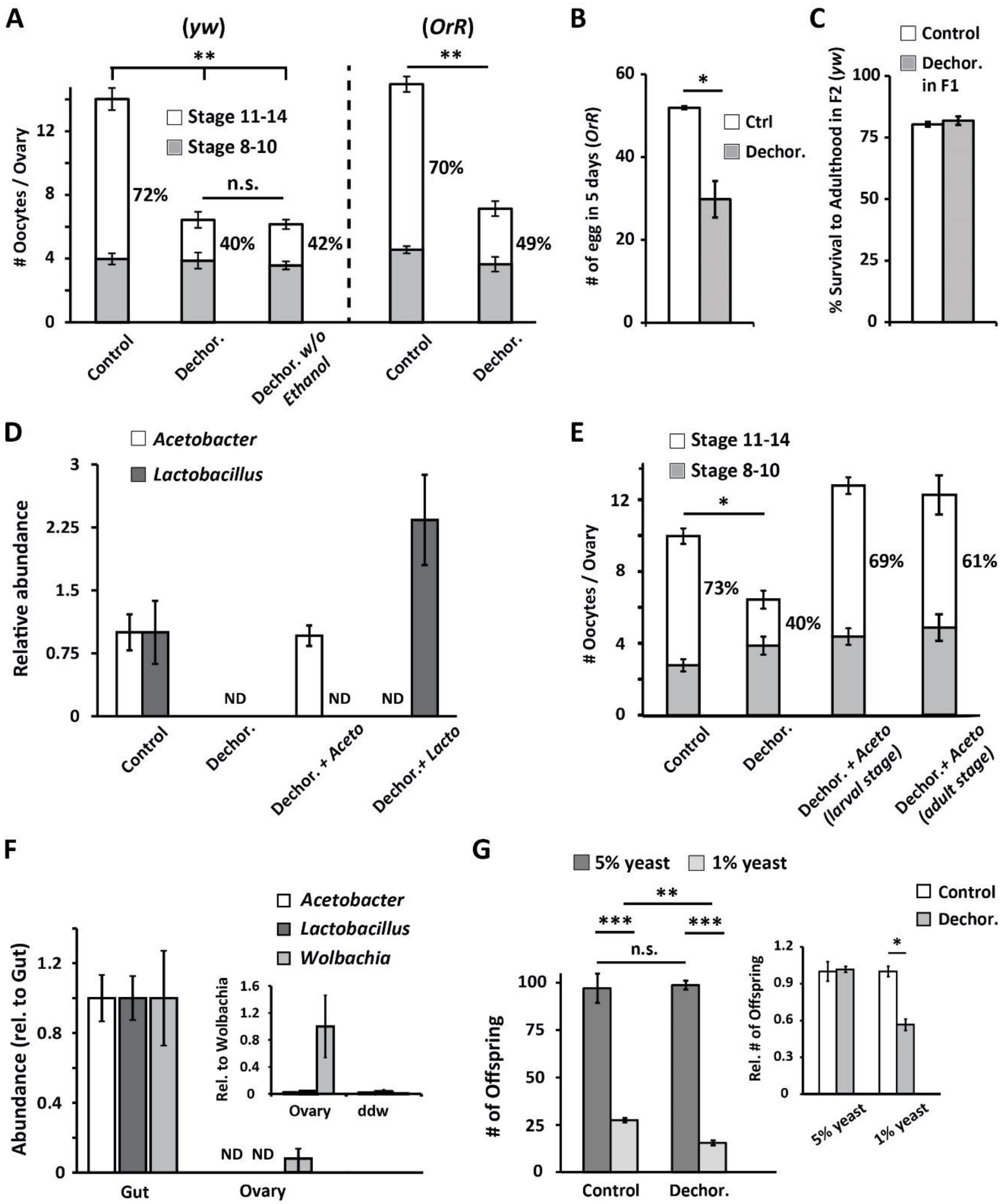

Supplementary Figure 1: Suppression of reproduction capacity by removal of extracellular gut bacteria. (A) Left: Number of oocytes per ovary and percentages of oocytes in stages 11-14 and 8-10 measured in 6-day old yellow white (*yw*) females developed after dechorionated with and without further egg sterilization by ethanol (Dechor. and Dechor. without ethanol, respectively). Right: Same for females from a

Wolbachia-free Oregon-R (*OrR*) strain, with and without egg dechorionation. Mean ± SE, based on 3 replicated experiments each including over 20 ovaries. **(B)** Total number of eggs per female (*OrR* strain) deposited over 5 days. Mean ± SE, based on 3 replicated experiments each including ≥7 individual females. **(C)** Survival to adulthood of embryos of bacterial-depleted flies (Dechor. in F1) and embryos of untreated flies (Control). Mean ± SE. **(D)** Efficiency of bacterial recolonization in the gut of 3rd instar larvae after egg dechorionation and supplementation of *Acetobacter* and *Lactobacillus* species (from *Colony 1* and *Colony 7*, respectively [1]) to the larval food (initial amount of bacteria in each case was the same). Abundance of the *Acetobacter* and *Lactobacillus* was measured by qPCR using species-specific probes against 16S rDNA. Mean abundance relative to untreated larvae (Control) ± SE based on 3 biological replicates. 'ND' – Non-detected. **(E)** Same as (A) for: Untreated *yw* females (Control) and *yw* females developed from dechorionated eggs (Dechor.), with and without supplement of *Acetobacter* at the embryo stage (Dechor. + *Aceto* (larval stage)) or after eclosion (Dechor. + *Aceto* (adult stage)). Mean ± SE, based on 3 replicated experiments, each with over 20 ovaries. **(F)** Relative abundance of *Acetobacter*, *Lactobacillus* and *Wolbachia* in the ovary of 3-6 day old *yw* females and the gut of 3rd instar *yw* larvae. Mean relative abundance ± SE compared to the levels of each bacterial species in the larval gut; based on 3 biological replicates. **Inset:** Relative abundance of *Acetobacter* and *Lactobacillus* species in the ovary and in a negative control sample (ddw). Mean relative abundance ± SE compared to the measured levels of *Wolbachia* in the ovary. **(G)** Effect of increasing dietary yeast (from 1 to 5%) on the absolute and relative (inset) numbers of eggs deposited by bacterial-depleted (Dechor.) and untreated females (Control). Mean ± SE based on 3 biological replicates. * $p < 0.05$, ** $p < 0.01$, *** $p < 0.001$ (Student's t-test, one-sided).

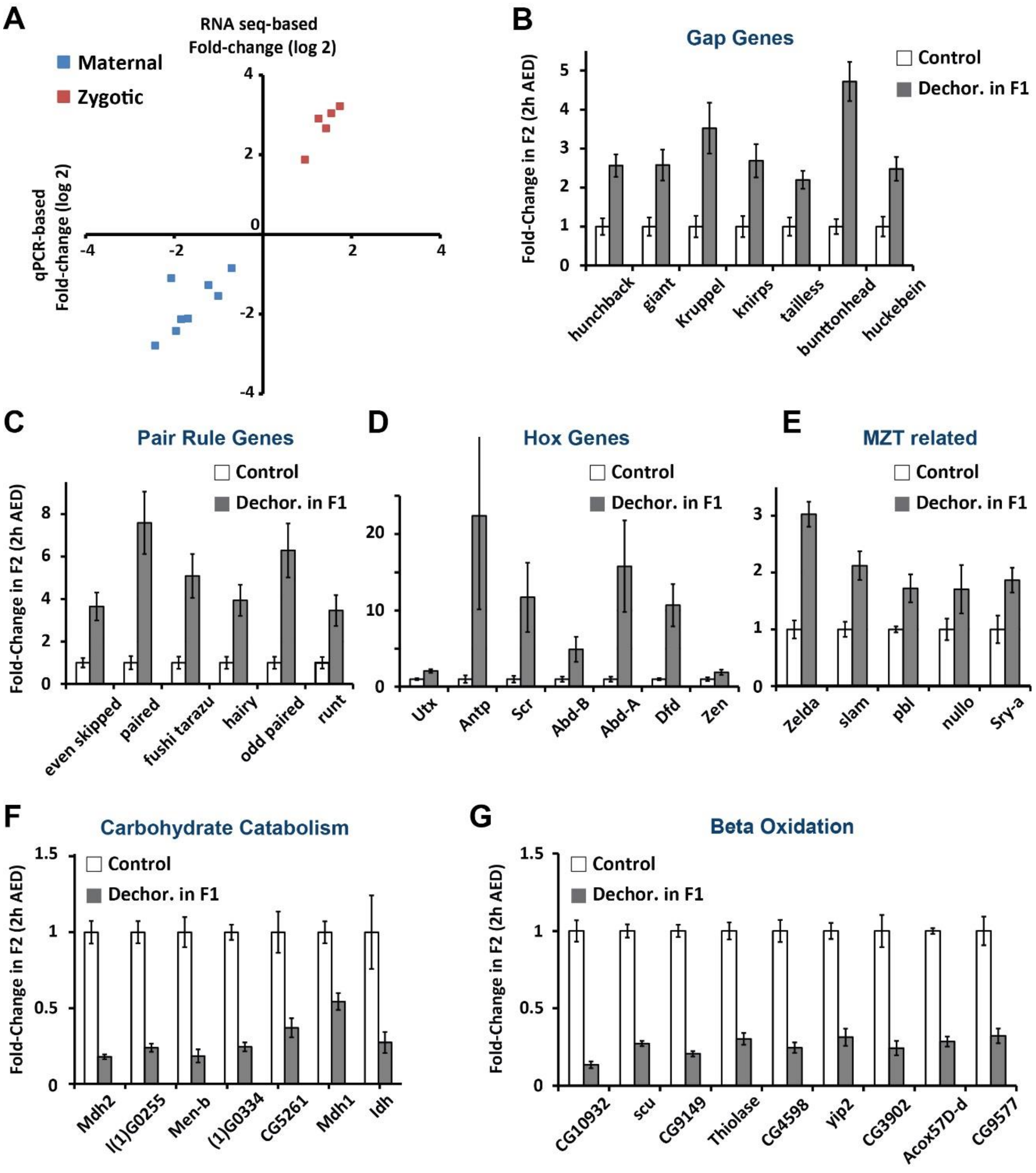

Supplementary Figure 2: qPCR verification of RNA-seq results for a representative set of maternal and zygotic genes. **(A)** Direct comparison between qPCR-based and RNA seq-based measurements of fold-change. Mean qPCR-based fold-change ± SE in 3 biological replicates (*yw* strain). Mean RNA seq-based fold-change ± SE based on 6 independent measurements per condition (3 different wild-type lines, *yw*, Oregon-R and Canton-S, each measured by RNA-seq in biological duplicates). **(B-E)** Increase in transcript levels of representative Gap genes (B), Pair-Rule genes (C), Hox genes (D) and

MZT-related genes (E), all determined by RNA-seq in (2hr AED) embryos of bacterial-depleted (Dechor.) and untreated flies (Control). Mean fold-change vs. control ± SE based on 6 independent measurements per condition as in (A). **(F, G)** Decrease in transcript levels of enzymes required for the catabolism of carbohydrates (F) and lipids (G), determined by RNA-seq as in (B-E).

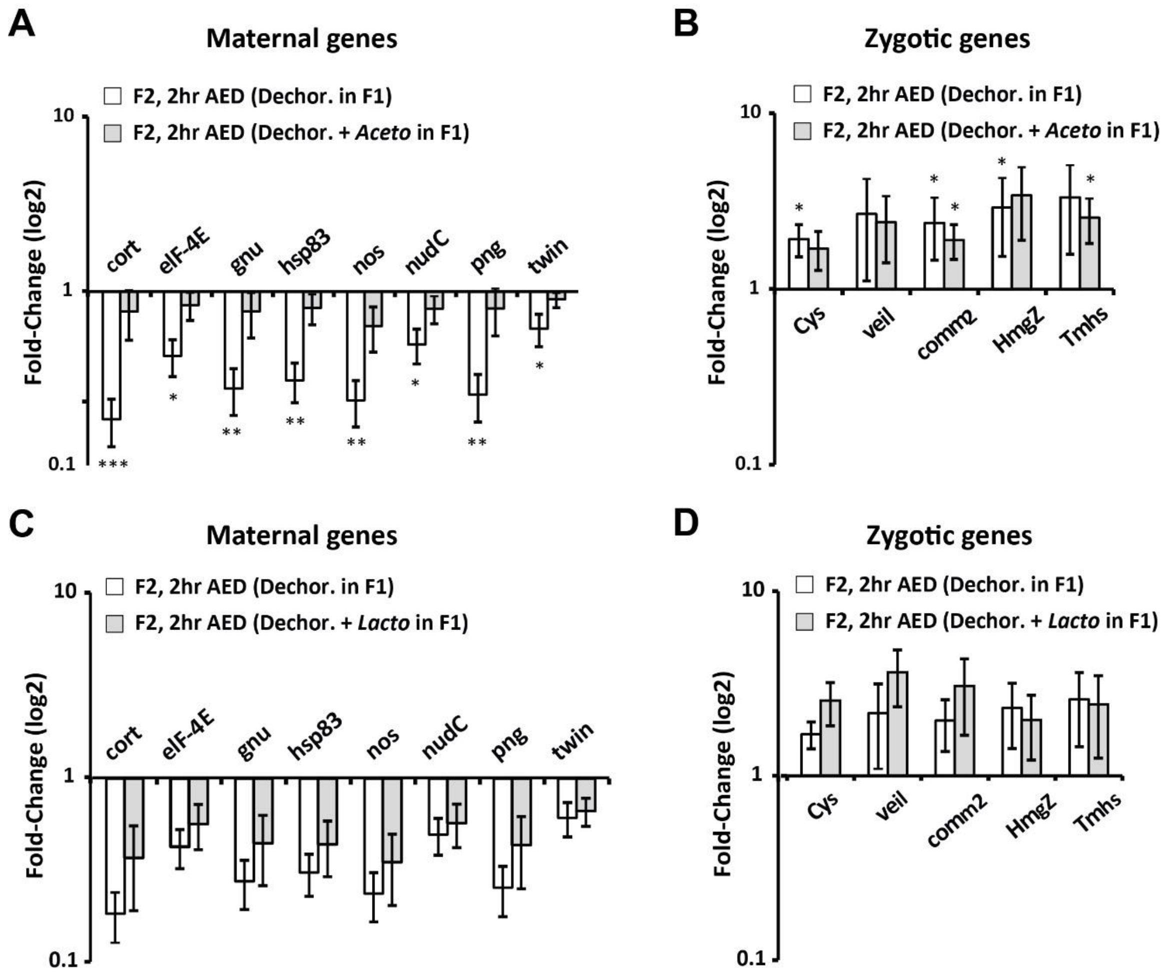

Supplementary Figure 3: Effects of species-specific specific bacterial repopulation on the levels of maternal and zygotic transcripts in the following generation of embryos (2hr after egg deposition). (A,B) Transcript fold-change in F2 embryos of F1 flies that were developed from dechorionated eggs, with and without supplementation of *Acetobacter* species (*Colony 1* [1]) in the larval food. Mean fold-change (qPCR-based) vs. control ± SE, n=3 * $p < 0.05$, ** $p < 0.01$ (Student's t-test). **(C,D)** Same as (A,B) for supplementation of *Lactobacillus* species (*Colony 7* [1]).

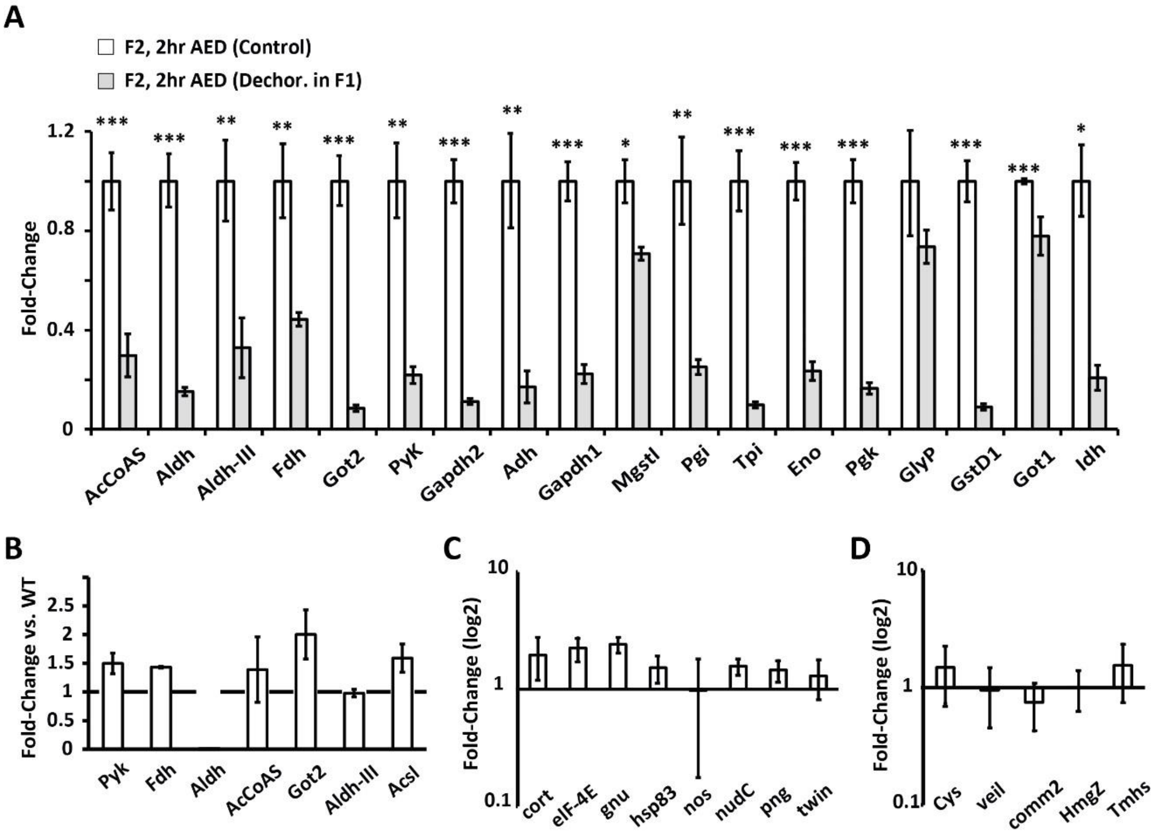

Supplementary Figure 4: *Aldh* downregulation alone does not reproduce the effect of bacterial depletion of embryogenesis in the next generation (A) Extension of Fig. 3B to additional genes within the *Aldh* network. Mean fold-change ± SE. ** $p < 0.01$, *** $p < 0.001$ (Wald Statistics, DESeq package[2]). **(B-D)** The effect of *Aldh* knockdown on transcript levels of representative *Aldh* network genes (B), maternal genes (C) and zygotic genes (D), measured by qPCR in 2hr embryos of *Aldh* null line ($Aldh^{24K}$) vs. wild-type embryos (*yw* strain). Mean fold-change ± SE in 3 biological replicates.

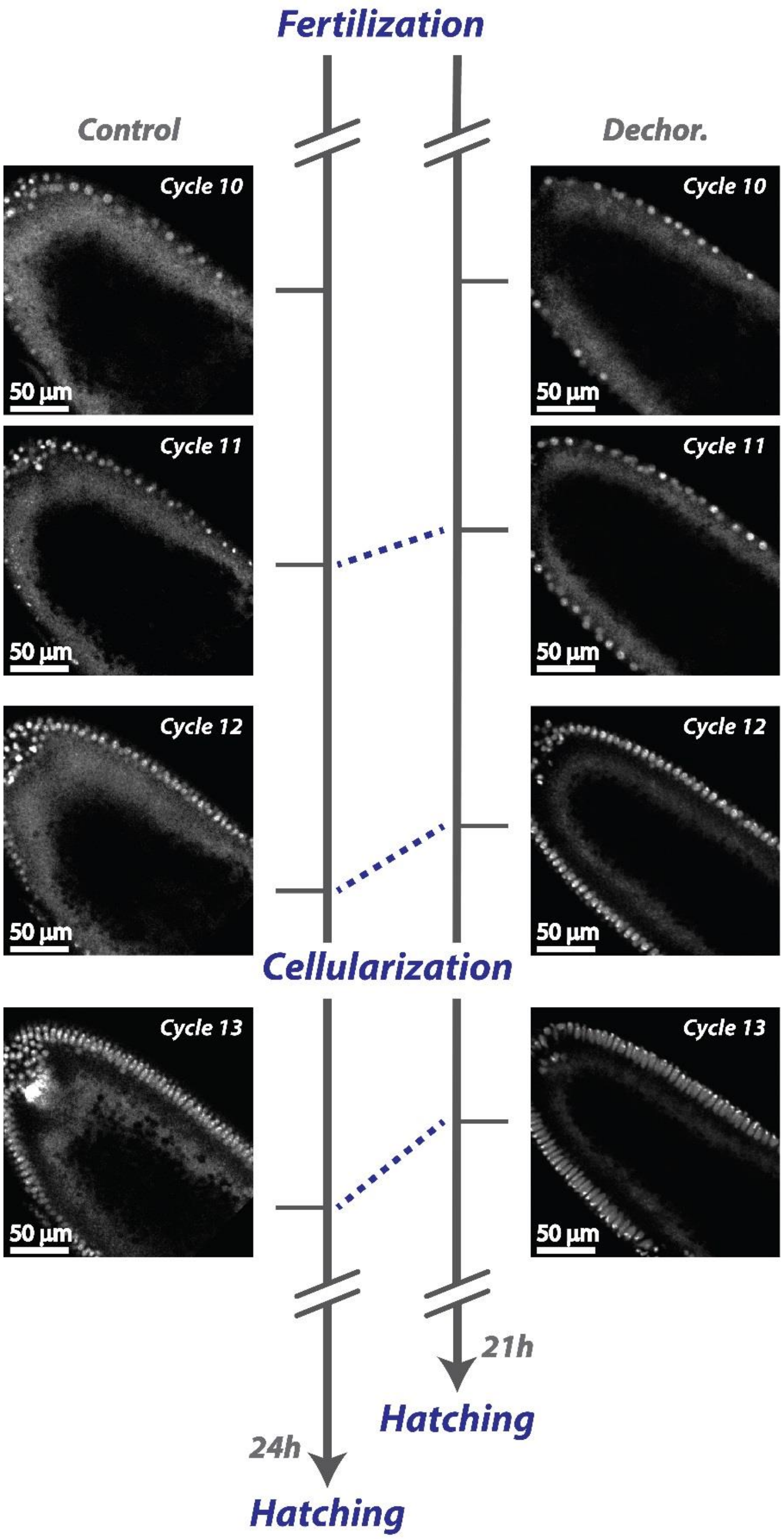
Fertilization
Control
Dechor.
Cycle 10
Cycle 11
Cycle 12
Cycle 13
50 μm
Cellularization
21h
Hatching
24h
Hatching

Supplementary Figure 5: Faster development of embryos of dechorionated parents vs. their control counterparts.

Sequence of images taken from a representative time-lapse confocal microscopy of *His2Av-mRFP* embryos [3], with (Dechor.) and without (Control) removal of extracellular gut bacteria in the preceding generation. The images span development cycles 10-13 (until the onset of cellularization; indicated) and show the beginning of the cycle. Gray lines in the middle represent the whole span of embryonic development with several stages (fertilization, cellularization, hatching) indicated. The skewed blue lines symbolize the emerging and increasing time difference between the onsets of corresponding cycles in the two conditions.

Supplementary Methods

RNA-seq analysis

Adaptors were removed from sequence reads using the cutadapt program [4, 5.] Reads were mapped to the drosophila transcriptome (Ensembl version BDGP.25) using Bowtie2 and TopHat software [6], then Cufflinks and Cuffmerge [6] were applied to define a list of transcripts that are comparable between all samples. Differentially expressed transcripts including fold-change and statistics were identified by applying two different methods. First Cuffdiff [6] was used directly on cufflinks output. The second method was to use the DESeq R package [7] on the bowtie2 output. Finally both methods were merged.

GO enrichments were computed using the 'David' online resource [8, 9, 10, 11] with cutoffs for up/down regulation and FDR set to 2-fold and 0.05, respectively. Up- and down-regulated gene-sets were analyzed separately.

Estimating developmental stage based on the transcriptional profile

The developmental stage of embryos at 2hr AED (with and without bacterial removal the in preceding generation) was estimated by comparing the RNA-seq mRNA data to a reference time-series transcriptional data from Lott et al. [12]. The estimation was based on the method developed by Efroni et al. [13] with a few modifications. The estimation requires identification of a subset of genes with a single peak of expression which is preceded and followed by a monotonic change along the reference time-series. This subset is used as a ruler for estimating the developmental stage of a query sample by determining, for each gene, its location along the time-series based on its measured expression in the sample. The stage of the sample as a whole is then determined by averaging the time estimations for all the genes in the subset. We adjusted the procedure to allow work with RNA-seq data and used two different schemes for estimating the stage: The first employed a stringent reference set of monotonically decreasing maternal genes (Supplementary Data 2) used as in Efroni et al. This reference

was defined by selecting from Lott et al.[12] all the genes which satisfy the criteria in Efroni et al., and narrowing this list by intersecting it with curated experimental maternal data from Thomsen et al.[14]. The second scheme was based on representing the time course data of Lott et al.[12] as a set of time varying vectors, each comprising averaged expression data for a particular time point. For every vector of average transcript levels in the sample, we computed the Euclidian distance from all the reference vectors in the time series data. The stage of each sample was then estimated by identifying the two reference vectors which correspond to the lowest distance from the sample and interpolating the stage between them based on the relative distance from them.

The estimations by the two schemes yielded very similar stages (no more than half a cycle difference). In this paper we used the estimation based on the first scheme and verified the main findings using the second scheme. All estimations were performed using in-house MATLAB scripts (available from the online github repository at https://github.com/elgartmi/AgeByRNA).

Inferring the *Aldh* network

The *Aldh* network of Fig. 3B was inferred from the literature using the STRING online resource [10]. STRING uses literature mining to infer connections between genes and assigns confidence to each interaction. We used it to define the *Aldh* network as follows: The highest confidence genes with direct connection to *Aldh* were determined using *Aldh* as an input gene and selecting the top 10 genes which are not defined only by a CG designation. Each of these genes was then re-fed into STRING to obtain secondary interactions, filtered in the same way as the genes with direct connection. To generate a connected map, STRING was queried the combined set of genes. The map was trimmed by removing secondary-interacting genes which have less than 2 interactions with the top 10 genes with direct connection to *Aldh*.

Measuring enzymatic activity of Aldh

Aldh activity was assayed in ovaries and larvae homogenates by monitoring the change in absorbance at 340nm in the reaction mixture as described by Moxon et al. [15]. 10 whole 3rd instar larvae or 15 dissected ovaries were homogenized, respectively, in 100μl or 20μl of extraction buffer described by Heinstra et al. [16]. Homogenates were allowed to stand for 15min on ice before centrifugation in a microfuge at 15,000g for 20min. The supernatants were used for subsequent assays and the operations were conducted at 4°C. Protein content was determined by the QBIT kit and used to standardize when comparing different samples. Aldh activity was measured with a TECAN device at 25 degrees Celsius. The absorbance at 340nm was recorded after addition of 15ul supernatant to 85ul of reaction buffer as described by Moxon et al. [17] containing acetaldehyde and NAD^+ (Sigma) supplemented with Pyrazole (to block ADH activity; Sigma) to the final concentration of 0.2M.

Supplementary References

1. Fridmann-Sirkis Y*, et al.* Delayed development induced by toxicity to the host can be inherited by a bacterial-dependent, transgenerational effect. *Frontiers in genetics* **5**, 27 (2014).

2. Anders S, Huber W. Differential expression analysis for sequence count data. *Genome biology* **11**, R106 (2010).

3. Di Talia S, She R, Blythe SA, Lu X, Zhang QF, Wieschaus EF. Posttranslational control of Cdc25 degradation terminates Drosophila's early cell-cycle program. *Current biology : CB* **23**, 127-132 (2013).

4. Martin M. Cutadapt removes adapter sequences from high-throughput sequencing reads. *EMBnetjournal* **17**, 10-12 (2011).

5. Werren JH, Baldo L, Clark ME. Wolbachia: master manipulators of invertebrate biology. *Nature reviews Microbiology* **6**, 741-751 (2008).

6. Trapnell C*, et al.* Differential gene and transcript expression analysis of RNA-seq experiments with TopHat and Cufflinks. *Nat Protoc* **7**, 562-578 (2012).

7. Anders S, Huber W. Differential expression analysis for sequence count data. *Genome biology* **11**, R106 (2010).

8. Huang DW, Sherman BT, Lempicki RA. Systematic and integrative analysis of large gene lists using DAVID bioinformatics resources. *Nature protocols* **4**, 44-57 (2009).

9. Huang DW, Sherman BT, Lempicki RA. Bioinformatics enrichment tools: paths toward the comprehensive functional analysis of large gene lists. *Nucleic acids research* **37**, 1-13 (2009).

10. Jensen LJ*, et al.* STRING 8--a global view on proteins and their functional interactions in 630 organisms. *Nucleic acids research* **37**, D412-416 (2009).

11. Laland K*, et al.* Does evolutionary theory need a rethink? In: *Nature* (ed^(eds) (2014).

12. Lott SE, Villalta JE, Schroth GP, Luo S, Tonkin LA, Eisen MB. Noncanonical compensation of zygotic X transcription in early Drosophila melanogaster development revealed through single-embryo RNA-seq. *PLoS biology* **9**, e1000590 (2011).

13. Efroni I, Blum E, Goldshmidt A, Eshed Y. A protracted and dynamic maturation schedule underlies Arabidopsis leaf development. *The Plant cell* **20**, 2293-2306 (2008).

14. Thomsen S, Anders S, Janga SC, Huber W, Alonso CR. Genome-wide analysis of mRNA decay patterns during early Drosophila development. *Genome biology* **11**, R93 (2010).

15. Moxon LN, Holmes RS, Parsons PA, Irving MG, Doddrell DM. Purification and Molecular-Properties of Alcohol-Dehydrogenase from Drosophila-Melanogaster - Evidence from Nmr and Kinetic-Studies for Function as an Aldehyde Dehydrogenase. *Comp Biochem Phys B* **80**, 525-535 (1985).

16. Heinstra PW, Geer BW, Seykens D, Langevin M. The metabolism of ethanol-derived acetaldehyde by alcohol dehydrogenase (EC 1.1.1.1) and aldehyde dehydrogenase (EC 1.2.1.3) in Drosophila melanogaster larvae. *The Biochemical journal* **259**, 791-797 (1989).

17. Eldh M*, et al.* Exosomes communicate protective messages during oxidative stress; possible role of exosomal shuttle RNA. *PloS one* **5**, e15353 (2010).
